# Loss and Return of Ticklishness in Functional Neurological Disorder

**DOI:** 10.1002/mdc3.14122

**Published:** 2024-06-09

**Authors:** Jan Adriaan Coebergh, Hassan Khalil, Arman Talwar, Mark Edwards, Francesca Morgante, Glenn Nielsen, Will Nash

**Affiliations:** ^1^ Department of Neurology St. George's Hospital NHS Foundation Trust London United Kingdom; ^2^ Department of Neurology Ashford/St. Peter's Hospital Chertsey United Kingdom; ^3^ Neurosciences and Cell Biology Institute, St George's University of London London United Kingdom; ^4^ Department of Clinical and Basic Neuroscience Institute of Psychiatry, Psychology and Neurosciences, King's College London London United Kingdom

**Keywords:** agency, dissociation, functional neurological disorder, sensory processing, ticklishness

## Abstract

**Background:**

People with functional neurological disorder (FND) have abnormalities in sensory processing. Loss of ticklishness has been rarely reported.

**Objectives:**

To describe associated clinical features in people with FND and loss of ticklishness and explore correlations with sensory changes.

**Methods:**

Retrospective audit of clinical letters of people diagnosed with FND in a tertiary clinic and further cases identified in a general neurology clinic.

**Results:**

Thirty‐eight patients with loss of ticklishness are described, of which most had other functional sensory symptoms and signs. It was more often localized to one limb, rather than generalized, in those with pain or weakness. Dissociation for the affected body part was often described.

**Conclusions:**

Loss of ticklishness in FND is frequently described and offers insights into mechanisms of agency, sensory processing and interoception, which are known to be altered in FND.

Functional neurological disorder (FND) is common and frequently disabling. Current criteria stress establishing a diagnosis based on positive findings from history and examination.

Sense of agency, interoception and sensory processing (including sensory attenuation) have been shown to be altered in FND.^1^

Ticklishness is a sense that contains a light, unpleasant component called “kinesmesis”,^2^ and a heavier, laughter‐associated component called “gargalesis.”^3^ It is a complex sensation involving stimulation of various mechanoreceptors^4^ and anticipation alone can lead to a response,^5^ something also seen in FND.^6^ It has been theorized that ticklishness is an evolutionary protective response to any foreign object moving or brushing up on you. Since most humans cannot tickle themselves, there is likely an important role for agency.^7^ To put it simply, if your body/a body part does not feel part of you (which people with FND commonly describe) you are unlikely to be ticklish in it.

Here we hypothesized that people with FND might manifest loss of ticklishness in the course of their condition, something only once previously briefly mentioned in the medical literature.^8^ In the present study, we explored this clinical phenomenon and its relationship to sensory signs and symptoms in a cohort of subjects with FND.

## Methods

A retrospective audit of clinic letters was conducted between January 1, 2018 and March 31, 2021 from a general neurology clinic in a district general hospital and from a tertiary national FND clinic, where clinic letters of 136 people with FND were examined. Clinical documentation of patients presenting with FND to one author (JC) was reviewed. Patients were included in the study if they reported loss of ticklishness spontaneously or on direct questioning; it was not assessed in every patient seen. Ticklishness was assessed by asking, “Are you as ticklish here as you are there,” as soft stimuli were applied to their feet. We attempted to avoid suggestion or leading the patient to any particular answer. Loss of ticklishness was reported as generalized or localized. In each subject we identified the type of FND (motor, sensory, cognitive, mixed) and its phenomenology (ie, weakness, involuntary movements, paroxysmal epileptic seizures). We also retrieved information about presence of pain, other neurological or systemic disorders and dissociative experiences.

## Results

We identified 38 patients with FND who acknowledged loss of ticklishness (23 from the tertiary and 15 from the general clinic). Thirty‐four of the patients were female (89%). Thirty‐two patients (84%) had a localized loss of ticklishness and 6 (16%) had a generalized loss of ticklishness. Localized loss was more common with weakness in the associated limb (generally leg) and in those with a change in pain sensation. Sixteen patients were followed up and three (19%) reported a return of ticklishness. The three patients who reported return of ticklishness, had localized loss only and also saw improvement in their motor symptoms (fixed dystonia and weakness) (Table 1).

**TABLE 1:** Thirty‐eight total patients with loss of ticklishness were identified, 32 with localized loss of ticklishness and six with generalized loss of ticklishness

	Localized	Generalized
Total number	32	6
Altered/loss of sensation	21	4
Subjective loss of sensation	14	2
Objective loss of sensation	7	2
Change in Pain sensation	17	6
Loss of temperature sensation	4	1
Dystonia	3	2
Tremor	8	1
Spasms	7	1
Jerks	11	2
Mixed movement disorder	0	1
Weakness in affected limb	25	2
Fixed functional dystonia	3	3
Gait disorder	25	6
Body part dissociation	11	4
Return of ticklishness	3	0

Within these two subgroups the frequency of several neurological symptoms/signs were identified (eg, 8 patients who had localized loss of ticklishness experienced a tremor).

Of the 136 people with an FND diagnosis in the tertiary clinic, 62 mentioned experiencing other sensory symptoms (18 with positive phenomena and 21/44 of those with negative phenomenona had abnormalities on the sensory examination); 23 of the 62 identified had loss of ticklishness.

Twenty‐five patients (66%) reported reduced or altered sensation in the area where there was a loss of ticklishness. Amongst this subgroup, 16 (64%) patients had a subjective change of sensation to touch that did not follow an anatomical sensory distribution; 20 (80%) reported retained temperature sensation; and five (20%) described change of both temperature and touch. Twenty‐two patients (58%) had pain in the affected limb. Thirteen patients had normal sensation, and no pain, despite having a loss of ticklishness.

Dissociation for a body part was described by 15 (39%) patients. The descriptions varied, but most described a feeling that the body part does not belong to them. In some of the cases, this feeling was intermittent and lasted between 20 s and several days, while the feeling was permanent for others. One patient had brief attacks where she could see herself from above (autoscopy) (extensive investigations for epilepsy normal).

Thirty‐three (79%) had a previous diagnosis of depression or anxiety and/or lifetime adversity. Thirty patients (79%) had co‐morbid neurological conditions, most commonly migraine and epilepsy. Twenty‐three (61%) of the 38 patients had both neurological and psychiatric/social perpetuating factors.

In 37 of the 38 patients’ data on laterality of loss of ticklishness was collected. Twelve patients were identified to have loss of ticklishness lateralised to the left side of the body, 12 to the right side of the body and 13 having loss of ticklishness in both sides of the body.

## Discussion

In this retrospective study we found 38 people diagnosed with FND who also reported loss of ticklishness. The pathophysiology of functional neurological disorder (FND) is often explained by a predictive coding model. This can integrate interoceptive, motor and social aspects of emotion,^9^ something which ticklishness is a prime example of. Predictive processing models of FND are consistent with excessive “top down” influences on upcoming sensory input, which would predict that afferent signals, including those mediating ticklishness, would be down‐weighted. “Kinesmesis,” the soft and itchy tickle, is relayed by the nerve fibers that conduct light touch through the ventral spinothalamic tracts (so should often co‐exist with changes to pain perception), whereas, “gargalesis,” the laughter associated tickle is caused by stimulation of pressure‐sensitive areas by application of high pressure and are relayed by fibers that conduct deep touch, proprioception, vibration through the dorsal columns.^10^ The dissociation of pain perception and ticklishness in many of our patient group is therefore inconsistent with known peripheral and spinal cord anatomy, suggesting that higher order processing is relevant. The peripheral input is processed in the somatosensory cortex with inputs from the anterior cingulate gyrus, salience network, prefrontal cortex (VMPFC and DLPFC) and the TPJ. The laughter component of tickling involves the anterior SMA. Using functional neuroimaging studies, Blakemore showed the involvement of the ACC and sensory attenuation in tickling.^6^ In FND, altered activation of the ACC, VMPFC,^11^ SMA, DLPFC, TPJ and angular gyrus are also seen.^12^

It is known most patients with FND have sensory abnormalities and symptoms. Loss of ticklishness fits with low registration described in FND, which was correlated to anxiety.^13^ Subjects with continuous functional dystonia have normal pain thresholds and increased thresholds for unbearable pain (namely pain tolerance) in keeping with a dissociation between the sensory‐discriminative and cognitive‐emotional components of pain.^14^

Impairments in interoception,^15^ attentional allocation,^16^ and perceptual inferences^17^ are now increasingly being included in models of FND. A recent study in FND found higher susceptibility to dissociation which was related to lower interoceptive accuracy (awareness of normal physiological conditions of their body).^18^ This implies a lack of agency. Motor FND patients have a reduced sense of agency.^19^

The loss of ticklishness and dissociation for the affected body part are possibly clinical manifestations of this loss of agency. Body Integrity Identity Disorder and Xenomelia are two other conditions where people describe a body part does not belong.^20^

Ishiyama and Brecht found in rats in an anxiogenic scenario, “fear” suppressed the neuronal responses to tickling in the somatosensory cortex.^21^ Many patients with FND have co‐morbid anxiety and this was common in our case series. There are no human studies looking at the relationship between anxiety and loss of ticklishness. This is a potential area for future research. In terms of laterality, there is evidence that ticklishness is felt more on the right side of the body^22^; we found no obvious difference in our cohort of patients.

We acknowledge the following limitations for our study. We did not use standardized assessment of pain/temperature/touch or systematic scales for dissociation and anxiety. The validity and reliability of our measure of ticklishness is unknown. This was a retrospective review of notes. We did not ask everyone with FND if they had loss of ticklishness. However often partners corroborated the change and when tested in multiple people with reported unilateral change of ticklishness this was invariably supported by an examination of ticklishness at the bedside. No data was collected about specificity (absence/loss of ticklishness in the normal/other neurological disorders population). The cohort was predominantly made up of patients with functional movement disorders. It should be explored if the findings are similar or different in other FND subtypes such as functional seizures and functional dizziness.

Loss of ticklishness has a diverse relationship to other clinical symptoms in FND. It can be localized or generalized and can change with clinical improvement. Altered sensation (particularly light touch and pain) is common but not a prerequisite and often inconsistent. Exploring this symptom and its relationship to dissociation and anxiety may provide new insights into sensory disturbance, interoception and sense of agency in FND. Change in ticklishness could be useful to explain these disordered mechanisms to those affected.

## Author Roles

(1) Research Project: A. Conception, B. Organization, C. Execution; (2) Statistical Analysis: A. Design, B. Execution, C. Review and Critique; (3) Manuscript Preparation: A. Writing of the First Draft, B. Review and Critique

J.A.C.: 1A, 1B, 1C, 2A, 2C, 3A, 3B

H.K.: 1B, 1C, 2A, 2B, 2C, 3B

A.T.: 1C, 3A, 3B

M.E.: 1C, 3A,3B

F.M.: 3B

G.N.: 3B

W.N.: 3B

## Disclosures

**Ethical Compliance Statement:** Ethical compliance was confirmed through our Institutional Review Board. This work was registered as a clinical audit at St George's Hospitals (AUDI003911). We confirm that we have read the Journal's position on issues involved in ethical publication and affirm that this work is consistent with those guidelines. No informed consent was obtained. We confirm that we have read the Journal's position on issues involved in ethical publication and affirm that this work is consistent with those guidelines.

**Funding Sources and Conflicts of Interest:** No specific funding was received for this work and the authors declare that there are no conflicts of interest relevant to this work.

**Financial Disclosures for Previous 12 Months:** FM has received speaking honoraria from Abbvie, Medtronic, Boston Scientific, Bial, Merz; Travel grants from the International Parkinson's disease and Movement Disorder Society; Advisory board fees from Abbvie, Merz and Boston Scientific; Consultancies fees from Boston Scientific, Merz and Bial; Research support from NIHR, UKRI, Boston Scientific, Merz and Global Kynetic; Royalties for the book *Disorders of Movement* from Springer and is member of the editorial board of *Movement Disorders*, *Movement Disorders Clinical Practice*, *European Journal of Neurology*. JC has received speaking honoraria from Bial, Brittania, Merck advisory board fees and Teva; on patent for test of Gaba A antibodies; does medical expert reporting in personal injury and clinical negligence cases, including in cases of functional neurological disorder. GN receives research funding from the National Institute for Health and Care Research (NIHR). MJE does medical expert reporting in personal injury and clinical negligence cases, including in cases of functional neurological disorder. MJE has shares in Brain and Mind Ltd which provides neuropsychiatric and neurological rehabilitation in the independent medical sector, including in people with functional neurological disorder. MJE has received financial support for lectures from the International Parkinson's and Movement Disorders Society and the Functional Neurological Disorder Society. MJE receives Royalties from the Oxford University Press for his book: *The Oxford Specialist Handbook of Parkinson's Disease and Other Movement Disorder*. MJE receives grant funding, including for studies related to functional neurological disorder, from NIHR and the MRC. MJE is an associate editor of the *European Journal of Neurology*.
